# Differences in immune indicators among normal, high-risk, and esophageal cancer populations and development of a predictive model

**DOI:** 10.3389/fimmu.2026.1723700

**Published:** 2026-01-30

**Authors:** Jian Li, Shaoju Qian, Xu Yang, Chenchen Lv, Jiamin Zhang, Kaiwen Wang, Jiali He, Suli Wang, Yadi Liu, Zishan Yang, Zhili Chu, Jin Xia, Feng Ren

**Affiliations:** 1 The First Affiliated Hospital of Xinxiang Medical University, Xinxiang, China; 2 School of Basic Medical Sciences, Xinxiang Medical University, Xinxiang, Henan, China; 3 Department of Medical Oncology, Anyang Tumor Hospital & The Affiliated Anyang Tumor Hospital of Henan University of Science and Technology, Anyang, Henan, China; 4 Henan International Joint Laboratory of Immunity and Targeted Therapy for Liver-Intestinal Tumors, Xinxiang Medical University, Xinxiang, Henan, China; 5 Henan Research Center for Engineering Technology in Digestive Tract Tumor Immune Digital Decoding and Cell Therapy, Xinxiang Medical University, Xinxiang, China

**Keywords:** esophageal cancer, high-risk group, immune indicator, machine learning, predictive model

## Abstract

**Objective:**

To systematically compare the differences in immune characteristics among three populations (normal group, high-risk group, and esophageal cancer [EC] patients group) and construct a predictive model based on immune metrics, thereby exploring its value in EC risk assessment and early diagnosis.

**Methods:**

A total of 440 participants were enrolled, including 173 normal individuals, 162 high-risk individuals, and 105 EC patients. Peripheral blood samples were collected, and 75 immune metrics were detected using flow cytometry. First, 21 highly correlated indicators were eliminated through the Pearson correlation matrix (|r|≥0.7), leaving 54 indicators for further analysis. Multivariate analysis of variance (MANOVA) was used to assess the effect of EC grouping on immune metrics, adjusting for gender and age. Significant indicators were analyzed using analysis of covariance (ANCOVA) and the false discovery rate (FDR). Stratified sensitivity analysis was conducted to verify the robustness of the results. Finally, a predictive model was built using 12 key immune metrics, and 10 covariates. The model performance was evaluated by 10-fold cross-validation, neural network algorithm, accuracy, Kappa value, and area under the receiver operating characteristic curve (AUC).

**Results:**

MANOVA showed that EC grouping had a significant overall impact on the 54-immune metrics (Pillai = 0.585, *P* < 2.2×10^^−16^). After ANCOVA and FDR correction, 12 immune metrics with significant inter-group differences were identified (*P′* < 0.05), including Tcm CD4^+^ T cells, Naive CD8^+^ T cells, Tem CD8^+^ T cells, and FB CD8^+^ T cells. Stratified sensitivity analysis confirmed stable differences in key indicators (FB CD8^+^ T cells and Act NK cells) remained stable among subgroups. The neural network predictive model exhibited excellent discriminative efficacy: the overall cross-validation accuracy was 81.2%, the Kappa value was 0.709, and the AUC values for pairwise comparisons among the three populations were all higher than 0.90 (0.905 to 0.927).

**Conclusion:**

This study demonstrates distinct and stable immune profiles across EC risk groups. The immune-based machine learning model effectively differentiates normal, high-risk, and cancer populations, offering a promising non-invasive tool for early risk assessment and screening of esophageal cancer.

## 1 Introduction

Esophageal cancer(EC) is a common gastrointestinal malignancy worldwide, ranking high in both incidence and mortality, posing a serious threat to human life and health (1). According to statistics from the World Health Organization, the annual global death toll from EC exceeds 300,000. The situation is even more severe in China, where some areas exhibit a high-incidence trend, placing a heavy burden on the healthcare system and patients’ families (2–7).

The development and progression of EC is a complex, multifactorial, multistep process involving multiple genes. The progression from normal esophageal mucosa to precancerous lesions, then to early-stage and ultimately advanced esophageal cancer, often spans a considerable period (3, 8). Throughout this process, the body’s immune system plays a critical role. The immune system maintains homeostasis by identifying and eliminating abnormal cells, including tumor cells (4, 9). When immune function is normal, it can effectively suppress the proliferation and spread of tumor cells; however, once immune function becomes dysregulated or impaired, it may create favorable conditions for tumorigenesis and progression (4, 5, 10). Therefore, in-depth exploration of changes in immune status across different stages of EC is of great significance for understanding its pathogenesis, identifying effective early diagnostic biomarkers, and developing rational treatment strategies (11, 12).

In recent years, with continuous developments in immunology theory and technology, the detection of immune indicators has been widely applied in cancer research (13, 14). Previous studies have shown that the distribution and function of immune cell subsets undergo significant alterations in cancer patients—such as T-cell subset dysregulation and decreased natural killer (NK) cell activity—which are closely associated with tumor development, progression, prognosis, and treatment response (5, 6, 15). Although some studies have focused on the immune status of EC patients, most have only compared patients with healthy controls, lacking systematic analysis of the immune characteristics of high-risk group. As a potential group for EC development, high-risk individuals may already exhibit alterations in immune status. In-depth study of changes in immune indicators in this population could facilitate earlier detection of EC risk and provide a basis for early warning (16).

Furthermore, current research on immune indicators in EC often focuses on single or a limited number of markers, lacking comprehensive analysis of multi-dimensional immune parameters, which makes it difficult to fully reflect the overall immune status (5, 17). Moreover, how to use immune indicators to construct effective discrimination models to accurately distinguish between healthy individuals, high-risk groups, and EC patients remains a challenge (6, 18). An efficient predictive model could not only provide a powerful tool for early diagnosis but also assist clinicians in developing personalized treatment plans.

Based on the above context, this study aims to establish a standardized immunity database encompassing the normal group, high-risk group, and EC patients. A total of 75 immune indicators across these three groups will be systematically detected and analyzed, and significantly different immune markers will be screened out. Relevant covariates will be incorporated to construct a triple-classification prediction model. Through this research, differences in immune characteristics among populations with varying risks of esophageal cancer will be clarified, the value of immune indicators in EC risk assessment and diagnosis will be validated, and new insights and scientific evidence will be provided for the early warning, mechanistic research, and clinical treatment of esophageal cancer.

## 2 Materials and methods

### 2.1 Data source

The data of this study were derived from self-collected datasets involving three populations: normal group, high-risk group, and EC patients group. All data were collected through one-center field surveys and collaboration with medical institution. This was a single-center, single-institution study conducted under the primary oversight of Xinxiang Medical University in collaboration with the Anyang Tumor Hospital, which served as the primary clinical partner for patient recruitment and sample collection. For each participant, data were acquired via a combination of on-site questionnaire surveys, medical record extraction from healthcare facilities, and laboratory sample testing, covering demographic information, living habits, medical history, pathological diagnosis results, and immune indicator detection data. This data collection approach ensured the authenticity, completeness, and traceability of the data.

#### 2.1.1 Inclusion and exclusion criteria for normal group

The inclusion criteria were as follows: (a) From non-high-incidence areas and without a history of chronic digestive system diseases (such as gastroesophageal reflux disease, gastritis, etc.); (b) No family history of EC (family includes parents, children, siblings, grandparents, maternal grandparents) and no history of tumor diseases in the family; (c) No precancerous diseases or precancerous lesions of esophageal cancer; (d) No high-risk factors for EC including smoking and heavy drinking; (e) Aged ≥40 years without the aforementioned high-risk factors.

The exclusion criteria were as follows: (a) Having a family history of esophageal cancer; (b) Having precancerous diseases or precancerous lesions of esophageal cancer; (c) Having high-risk factors for esophageal cancer, such as smoking or heavy drinking; (d) Suffering from immune diseases or other conditions that may affect immune function; (e) Having received immunotherapy or used immune-related intervention drugs.

#### 2.1.2 Inclusion and exclusion criteria for high-risk group

The inclusion criteria were as follows: (a) Long-term residence in high-incidence areas of EC (such as Linzhou area, Anyang) (19); (b) Aged ≥40 years old or with a family genetic history (7); (c) Having high-risk factors for esophageal cancer, such as smoking, heavy drinking, head and neck or respiratory squamous cell carcinoma, preference for high-temperature and pickled food, and poor oral hygiene (20, 21); (d) No autoimmune diseases, other diseases that may affect immune function, or diabetes.

The exclusion criteria were as follows: (a) Normal group without the above high-risk factors; (b) Population diagnosed with precancerous lesions of EC or EC patients; (c) With a history of other tumors; (d) Suffering from immune system diseases or having received immunotherapy drugs.

#### 2.1.3 Inclusion and exclusion criteria for EC patients

The inclusion criteria were as follows: (a) Newly diagnosed patients without receiving other treatments; (b) Diagnosed as esophageal squamous cell carcinoma by histopathology or cytology, including EC patients in all clinical stages.

The exclusion criteria were as follows: (a) Normal group, high-risk group, or population with precancerous lesions without confirmed esophageal cancer; (b) With a history of other tumors; (c) Suffering from immune system diseases or having received immunotherapy drugs.

#### 2.1.4 Additional universal exclusion situations

Participants meeting any of the following conditions were excluded from all three populations: (a) Suffering from the following diseases; (b) Immune system-related diseases: Immunodeficiency diseases, severe combined immunodeficiency, acquired immunodeficiency syndrome; (c) Autoimmune diseases: Rheumatoid arthritis, systemic lupus erythematosus, multiple sclerosis, type 1 diabetes, thyroid autoimmune diseases, primary biliary cholangitis, primary sclerosing cholangitis, systemic sclerosis.

Using the following immune-related therapeutic drugs: (a) Anti-CD20 monoclonal antibodies (such as Rituximab), anti-CD38 monoclonal antibodies (such as Daratumumab), immune checkpoint inhibitors (PD-1 monoclonal antibodies such as Nivolumab, Pembrolizumab, Toripalimab, Sintilimab, Tislelizumab; anti-CD30 monoclonal antibodies such as Brentuximab Vedotin; bispecific T-cell engager antibodies such as Blinatumomab; anti-CD79b monoclonal antibodies such as Polatuzumab vedotin). (b) Autoimmune disease treatment drugs: Hormones (such as corticosteroids), immunosuppressants (including Azathioprine, Chlorambucil, Cyclophosphamide, Cyclosporine, Mycophenolate mofetil, Methotrexate), anti-inflammatory drugs (such as non-steroidal anti-inflammatory drugs (NSAIDs) and steroidal anti-inflammatory drugs (SAIDs), disease-modifying antirheumatic drugs (DMARDs, including chemical drugs, natural drugs, and biological agents), AK inhibitors (such as Tofacitinib and Baricitinib).(c) Allergic disease treatment drugs: Receptor blockers (first and second-generation antihistamines), mast cell stabilizers, anti-inflammatory corticosteroids, leukotriene inhibitors; immunotherapy for certain allergic diseases (such as atopic dermatitis); tumor immunotherapy drugs (PD-1/PD-L1 inhibitors such as Pembrolizumab and Nivolumab; CTLA-4 inhibitors such as Ipilimumab).

Having received the following immunotherapies: (a) Immune checkpoint inhibitor therapy (targeting CTLA-4, PD-1, PD-L1); (b) Molecular targeted therapy (targeting cell signal transduction pathways, proto-oncogenes and tumor suppressor genes, cytokine receptors, anti-tumor angiogenesis, suicide genes, etc.). (c)Adoptive immune cell therapy (including CAR-T, TIL, NK, CIK/DC-CIK). (d)Cytokine therapy (using cytokines or their receptors produced in the laboratory). (e)Tumor vaccines (including preventive vaccines and therapeutic vaccines such as Provenge/Cimavax). (f) Immunomodulator therapy (including biological response modifiers and immunosuppressants).

#### 2.1.5 Definition, sources, and operational verification of grouping criteria

To ensure the operational clarity, reproducibility, and generalizability of the study groups, this subsection details the key criteria used to define the Normal Control, High-Risk, and EC Patient groups. All criteria were established based on authoritative national and international guidelines and consensus statements. Explicit quantitative thresholds, verification procedures, and multi-center quality control protocols are described below.

##### 2.1.5.1 Operational definitions and authoritative sources

###### 2.1.5.1.1 Normal control group

Participants were classified into the Normal Control group if they met all the following criteria, designed to minimize exposure to known EC risk factors. Geographic risk was defined as having no history of residency (≥1 year) in recognized EC high-incidence areas, such as Linzhou, as per the national screening program definitions and ecological studies of high-risk regions (19, 22). Regarding behavioral risks, a lifetime smoking history of <100 cigarettes (CDC definition) was required (23). Alcohol consumption was assessed using a modified version of the Alcohol Use Disorders Identification Test (AUDIT-C) questionnaire (24), with thresholds set at an average daily ethanol intake of <40 g for men and <20 g for women, aligning with the World Cancer Research Fund (WCRF) recommendations for cancer prevention (25). The genetic risk criterion required the absence of any history of malignant tumors in first- and second-degree relatives, a standard practice in cancer epidemiology endorsed by major oncology guidelines (26, 27).

###### 2.1.5.1.2 High-Risk group

Participants were assigned to the High-Risk group based on the presence of one or more major risk factors derived from national screening guidelines and established epidemiological evidence. High-risk geographic exposure was stringently defined as continuous residence for ≥10 years in a designated high-incidence area (e.g., Linzhou), verified through household registration or consistent self-reporting (19, 22). A family genetic history required confirmation of EC in at least one first-degree relative, a key criterion in the Chinese Society of Clinical Oncology (CSCO) risk stratification (7, 27). For behavioral exposures, quantitative thresholds were applied: a smoking history of ≥10 pack-years (calculated as [cigarettes per day/20] × years smoked) based on IARC evidence for a dose-response relationship (28); heavy alcohol consumption defined as an average intake of ≥40 g/day for men and ≥20 g/day for women for a duration of ≥5 years, corresponding to the WCRF threshold associated with increased EC risk (28); a preference for very hot beverages/foods was defined as self-reported daily consumption (>1 time/day) of items perceived as “scalding” (estimated >65 °C), a habit classified as probably carcinogenic by IARC (29, 30); regular intake of pickled foods was defined as consumption ≥3 times per week for ≥10 years, based on meta-analyses of Chinese dietary patterns; poor oral hygiene was operationally defined by the presence of two or more of the following: brushing teeth <1 time/day, frequent gum bleeding (≥2 times/week), no professional dental cleaning in the past 3 years, or loss of ≥5 teeth not due to trauma, supported by case-control studies (31).

###### 2.1.5.1.3 EC patient group

Patients were included upon meeting the following strict criteria. The diagnostic gold standard required a confirmed histopathological diagnosis of esophageal squamous cell carcinoma (ESCC) according to the World Health Organization (WHO) Classification of Tumours of the Digestive System (5th edition) (32). All pathology slides were centrally reviewed by two senior pathologists. Disease staging included patients across all clinical stages (I-IV), classified according to the American Joint Committee on Cancer (AJCC)/Union for International Cancer Control (UICC) TNM staging system (8th edition) (33). To capture the pre-treatment immune landscape, patients must have been newly diagnosed and treatment-naïve, with a window of ≤8 weeks from pathological confirmation to study enrollment and no prior surgery, radiotherapy, chemotherapy, or targeted/immunotherapy, verified through hospital records.

##### 2.1.5.2 Verification of “No high-risk” status for the Normal Control group

A multi-layered approach was implemented to rigorously verify the absence of high-risk exposures in the Normal Control group. Primary verification was conducted through a standardized, pre-tested electronic questionnaire with built-in logic checks and consistency prompts (e.g., follow-up questions for negative answers on smoking). Secondary verification involved cross-referencing self-reported data with available medical or community health records for a randomly selected 20% of the control sample, focusing on chronic disease history and smoking status. Tertiary verification and quality control included a 100% manual review of all questionnaires by trained data managers to identify logical inconsistencies, followed by double data entry and software-assisted consistency checks, with discrepancies resolved by referring to source documents.

### 2.2 Immune indicators

For all participants meeting the inclusion and exclusion criteria, 5 mL of peripheral venous whole blood was collected by professional medical staff using vacuum blood collection tubes containing ethylenediaminetetraacetic acid (EDTA) anticoagulant under fasting conditions (8–12 hours of food deprivation). After collection, the blood tubes were gently inverted 5–8 times to ensure full mixing of the anticoagulant with blood and prevent blood coagulation. Samples were sent to the cooperative laboratory for processing, with the time from collection to processing less than 48 hours; if transportation was required, samples were stored at room temperature, with the transportation time not exceeding 4 hours, to ensure that the sample quality met the detection requirements.

The collected whole blood samples were used to detect 75 immune indicators, covering T cells and their subsets (e.g., helper T cells (Th cells), cytotoxic T cells (Tc cells), regulatory T cells (Treg cells)), NK cells and their subsets (e.g., mature NK cells, activated (Act) NK cells), gamma delta T (γδT) cells and their subsets (e.g., V-delta 1 (Vδ1) cells, V-delta 2 (Vδ2) cells), and B cells and their subsets (e.g., naive B cells, Plasma cells).

Flow cytometry (FCM) was used for detection, with a BD FACSCanto II flow cytometer and matching immune cell detection antibody kits (e.g., CD3/CD4/CD8 antibody panel, CD56/CD16 antibody panel) provided by BD Biosciences. The detection process was performed in strict accordance with the kit instructions and instrument operation specifications, and negative controls and positive controls were set for each batch of samples to ensure the repeatability and reliability of the detection results. The 75 immune indicators involved in this study and their abbreviations were detailed in **Supplementary Table S1**.

### 2.3 Statistical analysis

This study systematically investigated the association between EC grouping (normal group, high-risk group, and EC patients) and immune indicators. Data processing and analysis were performed using R software (Version 4.5.0).

Continuous data were presented as mean ± standard deviation (x ± s) or median (interquartile range) [M (Q25, Q75)], with inter-group comparisons conducted using analysis of variance (ANOVA) or non-parametric tests. Categorical data were expressed as counts (percentages) [n (%)], and inter-group comparisons were carried out using the chi-square (χ²) test.

First, a Pearson correlation matrix was constructed to identify and exclude 21 immune indicators with high collinearity (│r│≥0.7) (**Supplementary Figure S1**), and a total of 54 indicators were finally retained for subsequent analysis. On this basis, multivariate analysis of variance (MANOVA) with Pillai’s trace test was applied to evaluate the overall effect of EC grouping on immune indicators after adjusting for covariates such as gender, age, and marital status. For indicators showing significant overall differences, further individual tests were performed using univariate analysis of covariance (ANCOVA), and the false discovery rate (FDR) method was used to correct for multiple comparisons.

To verify the robustness of the results, stratified sensitivity analysis was further conducted. Stratification was performed by gender (male, female), age (<71 years, ≥71 years), educational level (primary school and below, junior high school, senior high school and above), and occupation (farmer, non-farmer). The aforementioned ANCOVA was repeated in each subgroup to test whether the association between immune indicators and population classification remained consistent.

In addition, to assess the discriminative ability of immune indicators for the three populations, 12 key immune indicators screened by ANCOVA were selected and combined with 10 covariates to construct a three-classification predictive model under a 10-fold cross-validation framework. Categorical variables were uniformly reclassified, a neural network (nnet) was used as the base algorithm, and hyperparameter optimization was performed via grid search (tuneLength = 5). Model performance was evaluated using cross-validated overall accuracy, Kappa coefficient, and area under the receiver operating characteristic curve (AUC), with a focus on the discriminative efficacy of pairwise comparisons among the three groups. The significance level of this study was set at α = 0.05, and a *P*-value < 0.05 was considered statistically significant.

#### 2.3.1 Neural network model details

A feedforward neural network for three-class classification was implemented using the `nnet` package in R 4.5.0, with strict adherence to reproducibility and anti-data leakage principles. To ensure full replicability, a fixed random seed (set.seed [123]) was set before model initialization. The model adopted a three-layer structure: an input layer with 22 nodes (12 key immune indicators + 10 covariates), a single hidden layer with 5 nodes (optimized via grid search over candidate values 3/5/7/9/11) using ReLU activation, and an output layer with 3 nodes corresponding to the three target classes. The output layer employed Softmax activation paired with multiclass cross-entropy loss to convert raw outputs into a probability distribution (sum of probabilities = 1) for class prediction.

Hyperparameters were tuned via 5-fold nested cross-validation to optimize model performance and generalization. The hyperparameter search grid included hidden layer node count (size: 3/5/7/9/11), weight decay coefficient (decay: 0.001/0.01/0.1/0.5/1.0), learning rate (0.01/0.05/0.1, indirectly controlled via nnet package parameters), maximum number of iterations (maxit: 500/1000/2000), and maximum allowable weights (MaxNWts: 500/1000/1500). The final optimized combination was determined as: size=5, decay=0.1, learning rate=0.05, maxit=1000, and MaxNWts=1000. Weight initialization followed the nnet default setting (normal distribution, mean=0, SD = 0.5). Training stopped when the reduction in cross-entropy loss was <1e-6 for 10 consecutive iterations, or when the maximum number of iterations was reached, whichever came first. No class weight adjustment or resampling techniques were used; instead, stratified 10-fold cross-validation was employed to ensure balanced class distribution across all training and validation subsets. All preprocessing steps were performed within each fold to avoid data leakage: continuous features were standardized using z-score (mean=0, SD = 1) calculated exclusively on the training subset of each fold, while categorical features were transformed via one-hot encoding.

Key packages used in the analysis are: nnet (model construction), caret (cross-validation and hyperparameter tuning), pROC (performance assessment), dplyr (data manipulation), and ggplot2 (visualization).

## 3 Results

### 3.1 Baseline characteristics analysis

A total of 440 participants were enrolled in this study, including 173 individuals in the normal group, 162 in the high-risk group, and 105 EC patients (**Table 1**). The age distribution was similar across the three groups, with a median (Q25, Q75) of 71.00 (67.00, 74.00) years in all groups (*P*>0.05). However, significant differences were observed among the three groups in terms of gender ratio, marital status, educational level, occupation, smoking status, drinking status, body mass index (BMI), physical activity, and family history of malignant tumors (all *P* < 0.05).

**Table 1:** Descriptive analysis for baseline characteristics of the study participants.

Characteristic	Normal group (n=173)	High-risk group (n=162)	EC patients (n=105)	*P*
Age (median [Q25, Q75])	71.00 (67.00,74.00)	71.00 (67.00,74.00)	71.00 (67.00,74.00)	
Gender (%)				0.014
Male	74 (42.8)	69 (42.6)	62 (59.0)	
Female	99 (57.2)	93 (57.4)	43 (41.0)	
Marital status (%)				0.002
Married	154 (89.0)	122 (75.3)	91 (86.7)	
Others	19 (11.0)	40 (24.7)	14 (13.3)	
Education (%)				<0.001
Primary school and below	97 (56.1)	77 (47.5)	82 (78.1)	
Junior high school	44 (25.4)	67 (41.4)	15 (14.3)	
Senior high school and above	32 (18.5)	18 (11.1)	8 (7.6)	
Occupation (%)				<0.001
Farmer	106 (61.3)	144 (88.9)	88 (83.8)	
Non-farmer	67 (38.7)	18 (11.1)	17 (16.2)	
Smoking status (%)				0.005
Non-smoker	150 (86.7)	141 (87.0)	77 (73.3)	
Smoker	23 (13.3)	21 (13.0)	28 (26.7)	
Drinking status (%)				0.001
Non-drinker	143 (82.7)	139 (85.8)	71 (67.6)	
Drinker	30 (17.3)	23 (14.2)	34 (32.4)	
BMI (median [Q25, Q75])	26.00 (24.03, 28.00)	23.85 (21.00, 27.00)	22.00 (20.00, 25.00)	<0.001
Physical activity (%)				0.001
Non-sweating	87 (50.3)	60 (37.0)	62 (59.0)	
Sweating	86 (49.7)	102 (63.0)	43 (41.0)	
Family history of malignancy (%)				<0.001
No	154 (89.0)	120 (74.1)	66 (62.9)	
Yes	19 (11.0)	42 (25.9)	39 (37.1)	
T cells (mean [SD])	65.32 (10.95)	63.24 (12.80)	62.24 (12.99)	0.092
Tc cells (mean [SD])	37.69 (11.80)	43.10 (10.40)	31.22 (10.80)	<0.001
DN T lymphocytes (mean [SD])	6.36 (5.21)	10.23 (5.01)	5.87 (5.05)	<0.001
Naive CD4^+^ T cells (mean [SD])	33.35 (14.53)	34.55 (13.06)	31.39 (14.88)	0.202
Td CD4^+^ T cells (mean [SD])	5.19 (5.77)	6.46 (5.73)	4.25 (6.61)	0.01
Tcm CD4^+^ T cells (mean [SD])	27.26 (8.55)	27.13 (7.90)	32.75 (11.09)	<0.001
Sen CD4^+^ T cells (mean [SD))	11.21 (10.42)	11.37 (9.67)	9.26 (7.86)	0.168
Naive CD8^+^ T cells (mean [SD])	12.35 (8.63)	7.45 (5.15)	8.97 (7.15)	<0.001
Tcm CD8^+^ T cells (mean [SD])	5.84 (4.93)	2.05 (1.38)	7.21 (5.13)	<0.001
Tem CD8^+^ T cells (mean [SD])	22.15 (16.31)	20.01 (9.38)	36.56 (15.16)	<0.001
VS-Td CD8^+^ T cells (Inactive) (mean [SD])	28.59 (19.12)	56.52 (17.58)	36.42 (26.24)	<0.001
Sen CD8^+^ T cells (mean [SD])	63.06 (16.23)	72.45 (10.97)	57.17 (17.91)	<0.001
Total mem CD8^+^ T cells (mean [SD])	31.13 (15.72)	28.18 (11.79)	34.12 (16.72)	0.005
Homing mem CD8^+^ T cells (mean [SD])	25.57 (11.86)	28.29 (12.05)	31.99 (16.13)	<0.001
Tsen CD8^+^ T cells (mean [SD])	37.32 (16.09)	41.63 (14.83)	29.46 (14.30)	<0.001
Tfh cells (mean [SD])	18.23 (5.94)	16.79 (4.91)	19.12 (6.75)	0.004
Tfh17 cells (mean [SD])	16.14 (7.92)	18.96 (6.84)	12.18 (6.52)	<0.001
Th1 cells (mean [SD])	32.68 (13.32)	39.63 (11.48)	24.78 (13.15)	<0.001
Th2 cells (mean [SD])	9.67 (4.55)	11.28 (7.73)	15.43 (6.59)	<0.001
Th17 cells (mean [SD])	3.40 (3.20)	4.75 (2.64)	2.74 (3.67)	<0.001
Th1/Th2 ratio	4.88 (4.99)	6.03 (5.28)	2.26 (2.41)	<0.001
Th17/Th2 ratio	0.51 (0.58)	0.76 (0.83)	0.22 (0.38)	<0.001
Tc1 cells (mean [SD])	35.80 (16.44)	30.63 (10.83)	34.06 (16.18)	0.005
Tc2 cells (mean [SD])	12.74 (13.24)	16.46 (8.72)	7.14 (7.31)	<0.001
Tc17 cells (mean [SD])	3.00 (3.94)	2.05 (2.50)	3.42 (4.06)	0.004
Tph cells (mean [SD])	6.26 (4.91)	9.58 (6.14)	4.00 (4.72)	<0.001
Act Tfh cells (mean [SD])	2.64 (1.64)	3.18 (1.78)	1.80 (1.43)	<0.001
T/NK cells (mean [SD])	5.64 (4.29)	5.73 (4.44)	5.85 (4.01)	0.927
NK cells (mean [SD])	20.21 (9.51)	17.51 (8.98)	18.13 (11.24)	0.033
Im NK/Mat NK ratio	0.04 (0.02)	0.05 (0.02)	0.03 (0.02)	<0.001
Early FB NK cells (mean [SD])	48.86 (19.40)	49.59 (18.57)	48.84 (19.81)	0.926
Late FB NK cells (mean [SD])	10.35 (9.26)	9.35 (8.37)	9.35 (6.97)	0.474
Act NK cells (mean [SD])	94.21 (5.10)	87.75 (8.91)	87.82 (11.11)	<0.001
Conv-cyto NK cells (mean [SD])	39.11 (18.66)	41.32 (18.40)	31.82 (19.82)	<0.001
VS cyto NK cells (mean [SD])	32.98 (15.77)	37.42 (17.97)	32.51 (22.50)	0.04
γδT cells (mean [SD])	6.19 (5.24)	6.71 (5.08)	5.54 (4.61)	0.178
Vδ1 cells (mean [SD])	31.52 (25.95)	28.13 (22.06)	32.84 (22.91)	0.234
Vδ2 cells (mean [SD])	45.31 (26.41)	35.98 (25.88)	42.66 (25.13)	0.004
Vδ1/Vδ2 ratio	3.70 (12.88)	7.51 (41.42)	4.45 (18.42)	0.431
Func mat Vδ2 cells (mean [SD])	96.32 (5.93)	97.25 (5.96)	98.17 (2.36)	0.017
FB Vδ2 cells (mean [SD])	8.77 (9.99)	4.24 (8.43)	10.80 (8.69)	<0.001
Conv-cyto Vδ2 cells (mean [SD])	1.35 (1.69)	1.98 (5.13)	0.92 (1.05)	0.033
VS Vδ2 cells (mean [SD])	2.61 (7.10)	1.75 (4.51)	2.07 (3.66)	0.351
Func mat Vδ1 cells (mean [SD])	93.84 (8.96)	92.50 (8.60)	98.18 (2.86)	<0.001
FB Vδ1 cells (mean [SD])	16.82 (16.86)	6.80 (10.16)	20.76 (16.49)	<0.001
Conv-cyto Vδ1 cells (mean [SD])	1.54 (2.34)	2.06 (3.51)	2.38 (7.66)	0.295
VS Vδ1 cells (mean [SD])	2.37 (3.31)	2.34 (4.48)	2.74 (2.83)	0.645
B cells (mean [SD])	8.93 (4.23)	9.11 (5.21)	8.30 (4.97)	0.388
MZ B cells (mean [SD])	9.49 (6.24)	8.36 (5.37)	8.02 (5.84)	0.078
CD21^−^ B cells (mean [SD])	7.62 (6.70)	9.05 (8.52)	9.47 (10.89)	0.148
Pre-naive cells (mean [SD])	0.29 (0.43)	0.34 (0.32)	0.58 (0.93)	<0.001
Plasma cells (mean [SD])	0.79 (0.66)	0.73 (0.58)	1.38 (1.87)	<0.001
Classical switched B cells (mean [SD])	18.39 (10.39)	18.31 (8.48)	17.32 (8.79)	0.617
Im Reg B cells (mean [SD])	2.36 (1.77)	2.69 (2.93)	1.94 (1.47)	0.028

Specifically, the EC patient group had a higher proportion of males (59.0%), smokers (26.7%), drinkers (32.4%), and individuals with a family history of malignant tumors (37.1%), along with the lowest BMI (22.21 ± 3.40) and the highest proportion of participants with educational level of primary school or below (78.1%). The high-risk group had the highest proportion of farmers (88.9%), the lowest proportion of married individuals (75.3%), and the highest level of sweating-type physical activity (representing moderate-to-high intensity activity, 63.0%). The normal group had the highest BMI (26.26 ± 4.53) and the highest proportion of participants with educational level of senior high school or above (18.5%).

Regarding immune cell levels, significant differences were observed among the three groups for most indicators (*P* < 0.05 or *P* < 0.001), while only a few indicators (e.g., T/NK cells, Early FB NK cells) had no significant differences (*P*>0.05). Among them, helper T cells, effector memory (Tem) CD8^+^ T cells, Th2 cells, mature NK cells, and Plasma cells were the highest in EC patients; Cytotoxic T cells, terminally differentiated CD8^+^ T cells, Th1 cells, and conventional cytotoxic (Conv-cyto) NK cells were the highest in the high-risk group; activated (Act) NK cells, B10 cells, and Vδ2 cells were the highest in the normal group; regulatory T cells were higher in EC patients than in the other two groups; and the total number of T cells was similar across the three groups (*P* = 0.092).

### 3.2 Analysis of inter-group differences in immune indicators

**Figure 1:**
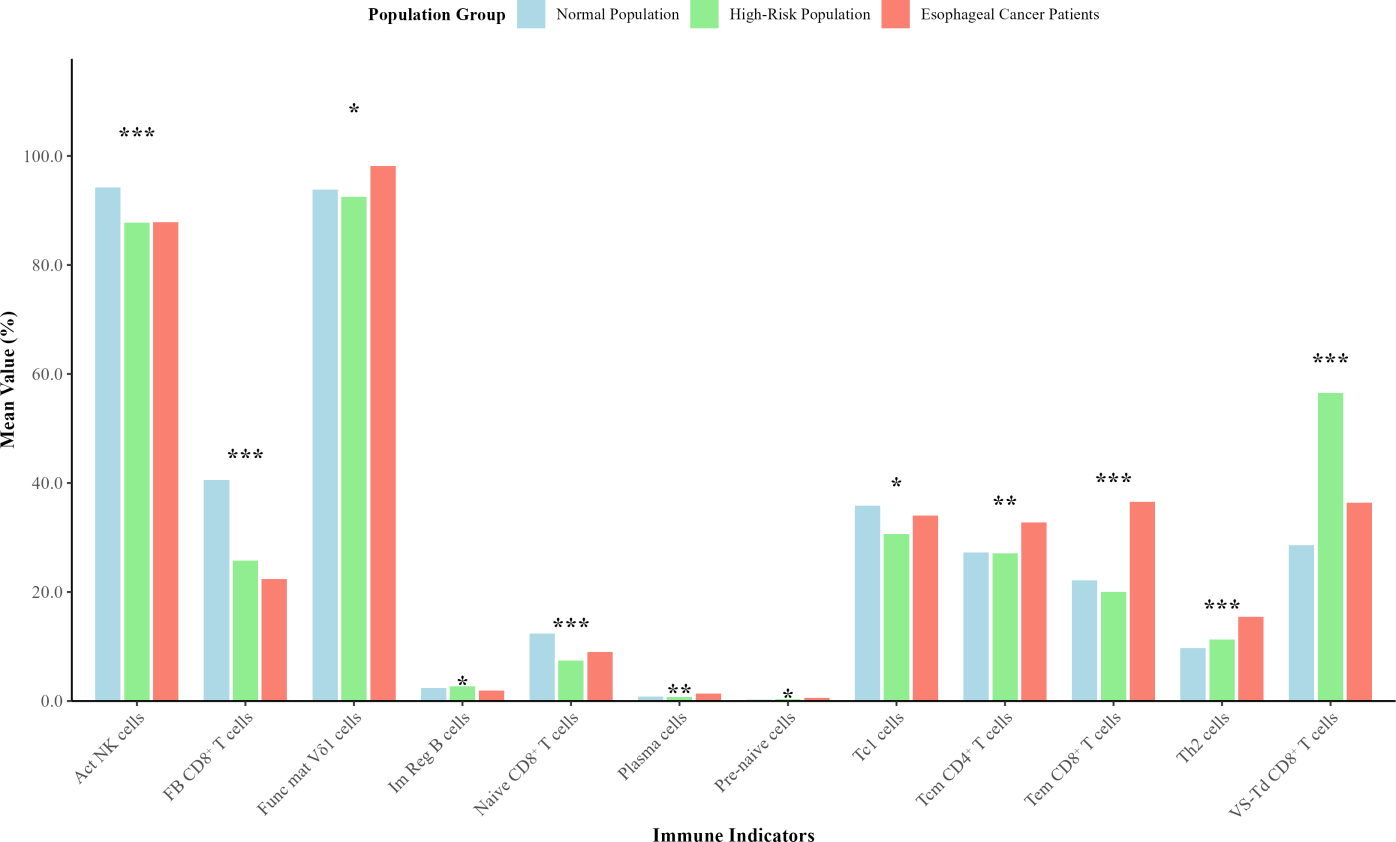
Bar chart of 12 meaningful immune indicators among three groups. ***P < 0.001, **P < 0.01, *P < 0.05.

MANOVA showed that EC grouping had a significant overall effect on the 54 immune indicators (Pillai = 0.585, *P* < 2.2×10^^−16^) (**Table 2**). Univariate analysis revealed that 12 immune indicators still exhibited significant inter-group differences after FDR correction (*P′* < 0.05) (**Figure 1**), including central memory (Tcm) CD4^+^ T cells, Naive CD8^+^ T cells, Tem CD8^+^ T cells, Virus-specific terminally differentiated (VS-Td) CD8^+^ T cells, functionally blocked (FB) CD8^+^ T cells, Th2 cells, Act NK cells, Plasma cells, functionally mature (Func mat) Vδ1 cells, immature regulatory (Im Reg) B cells, Pre-naive cells, and Tc1 cells. These indicators cover multiple aspects of cellular immunity, humoral immunity, and innate immunity. In addition, 3 indicators (Naive CD4^+^ T cells, Conv-cyto NK cells, and MZ B cells) showed marginal significance (*P* < 0.05, *P′* ≥ 0.05). The remaining 39 immune indicators had no statistically significant inter-group differences (*P′* ≥ 0.05) (**Table 3**).

**Table 2:** Multivariate analysis of variance (MANOVA) of immune indicators.

Analysis factors	*P*_Pillai_	F	*P*
EC Grouping	0.585	9.794	< 0.001
Gender	0.370	4.079	0.000
Age	0.349	3.727	0.000
Marital status	0.134	1.071	0.349
Education	0.175	1.475	0.021
Occupation	0.260	2.435	0.000
Smoking status	0.168	1.407	0.038
Drinking status	0.148	1.211	0.159
Physical activity	0.116	0.909	0.657
BMI	0.166	1.382	0.046
Family history of malignancy	0.099	0.761	0.890

**Table 3:** Analysis of covariance (ANCOVA) of immune indicators.

Immune metrics	F	*P*	*P’*	Immune metrics	F	*P*	*P’*
T cells	2.193	0.139	0.301	T/NK cells	0.113	0.737	0.904
Cytotoxic T cells	2.253	0.134	0.301	NK cells	2.325	0.128	0.301
DN T lymphocytes	1.197	0.275	0.511	Im NK/Mat NK ratio	4.633	0.032	0.101
Naive CD4^+^ T cells	5.422	0.020	0.073	Early FB NK cells	1.608	0.205	0.411
Td CD4^+^ T cells	0.033	0.857	0.922	Late FB NK cells	0.003	0.958	0.958
Tcm CD4^+^ T cells	14.735	0.000	0.001	Act NK cells	29.012	0.000	0.000
Sen CD4^+^ T cells	0.076	0.782	0.920	Conv-cyto NK cells	6.194	0.013	0.055
Naive CD8^+^ T cells	22.758	0.000	0.000	VS cyto NK cells	0.668	0.414	0.699
Tcm CD8^+^ T cells	0.021	0.885	0.922	γδT cells	0.042	0.837	0.922
Tem CD8^+^ T cells	25.128	0.000	0.000	Vδ1 cells	0.063	0.802	0.922
VS-Td CD8^+^ T cells	19.932	0.000	0.000	Vδ2 cells	0.928	0.336	0.585
FB CD8^+^ T cells	31.124	0.000	0.000	Vδ1/Vδ2 ratio	1.902	0.169	0.350
Sen CD8^+^ T cells	0.435	0.510	0.725	Func mat Vδ2 cells	2.482	0.116	0.285
Total mem CD8^+^ T cells	3.356	0.068	0.192	FB Vδ2 cells	0.277	0.599	0.830
Homing mem CD8^+^ T cells	3.059	0.081	0.208	Conv-cyto Vδ2 cells	0.006	0.940	0.957
Tsen CD8^+^ T cells	3.659	0.056	0.169	VS Vδ2 cells	0.502	0.479	0.703
Tfh cells	0.020	0.888	0.922	Func mat Vδ1 cells	8.486	0.004	0.017
Tfh17 cells	0.496	0.482	0.703	FB Vδ1 cells	0.181	0.671	0.883
Th1 cells	0.521	0.471	0.703	Conv-cyto Vδ1 cells	0.142	0.707	0.904
Th2 cells	18.393	0.000	0.000	VS Vδ1 cells	0.566	0.452	0.703
Th17 cells	1.150	0.284	0.512	B cells	0.515	0.473	0.703
Th1/Th2 ratio	1.501	0.221	0.427	MZ B cells	6.025	0.015	0.056
Tc1 cells	9.558	0.002	0.012	CD21^−^ B cells	4.726	0.030	0.101
Tc2 cells	0.126	0.723	0.904	Pre-naive cells	9.516	0.002	0.012
Tc17 cells	0.213	0.644	0.870	Plasma cells	12.717	0.000	0.003
Tph cells	0.027	0.869	0.922	Class-switched B cells	0.075	0.784	0.920
Act Tfh cells	3.149	0.077	0.207	Im Reg B cells	8.745	0.003	0.016

### 3.3 Results of stratified sensitivity analysis

Stratified sensitivity analysis indicated that most significant immune indicators remained stable across different subgroups.

Stratification by gender (**Supplementary Table S2**): FB CD8^+^ T cells showed significant differences in both male (F = 29.157, *P* < 0.001, *P′* < 0.001) and female (F = 29.826, *P* < 0.001, *P′* < 0.001); Act NK cells were also significant in males (F = 13.015, *P* = 0.0004, *P′* = 0.005) and females (F = 28.037, *P* < 0.001, *P′* < 0.001).

Stratification by educational level (**Supplementary Table S3**): FB CD8^+^ T cells were significant in the “primary school and below” group (F = 18.097, *P* < 0.001, *P′* = 0.001), “junior high school” group (F = 29.955, *P* < 0.001, *P′* < 0.001), and “senior high school and above” group (F = 13.542, *P* = 0.0006, *P′* = 0.031).

Stratification by occupation (**Supplementary Table S4**): This indicator (FB CD8^+^ T cells) was also significant in farmers (F = 18.342, *P* < 0.001, *P′* = 0.001) and non-farmers (F = 27.216, *P* < 0.001, *P′* < 0.001).

Stratification by age (**Supplementary Table S5**): FB CD8^+^ T cells showed significant differences in both the <71 years group (F = 30.078, *P* < 0.001, *P′* < 0.001) and ≥71 years group (F = 28.410, *P* < 0.001, *P′* < 0.001).

### 3.4 Results of predictive model performance

**Figure 2:**
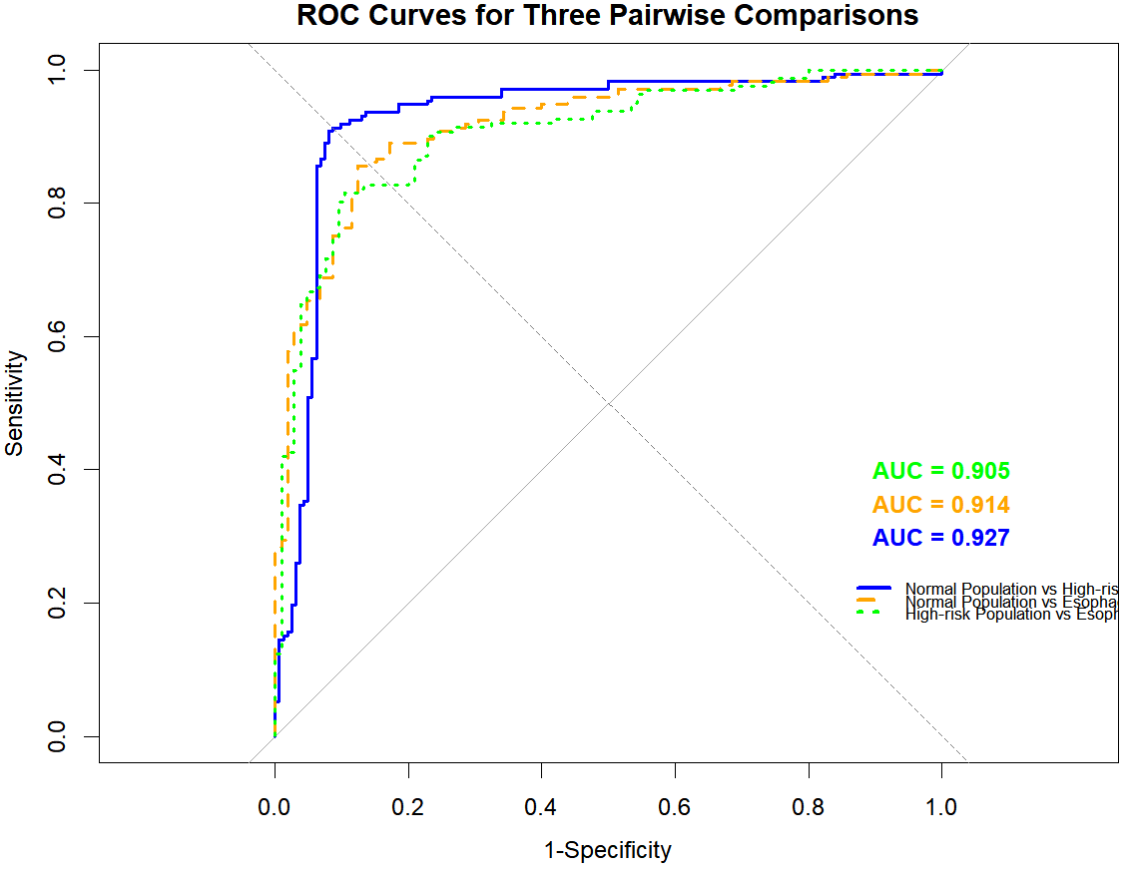
ROC curves for three pairwise comparisons.

A neural network predictive model was constructed based on immune indicators and covariates, and validated using 10-fold cross-validation, with the optimal hyperparameter combination identified as size = 5 and decay = 0.1. This model exhibited good overall discriminative efficacy for the three population categories, with a cross-validated overall accuracy of 81.2% and a Kappa coefficient of 0.709. The model showed excellent discriminative ability between each pair of categories (**Figure 2**): the AUC for the comparison between the normal group and high-risk group was 0.927, the AUC for the normal group vs. EC patients was 0.914, and the AUC for the high-risk group vs. EC patients was 0.905.

## 4 Discussion

### 4.1 Baseline characteristics differences and their association with EC risk

The normal group, high-risk group, and EC patients included in this study exhibited regular differences in baseline characteristics related to EC risk. age was evenly distributed across the three groups, with a median (Q25, Q75) of 71.00 (67.00, 74.00) years in all groups (*P*>0.05), which excluded age as a core confounding factor. However, significant differences (all *P* < 0.05) were observed among the three groups in terms of gender, marital status, education, occupation, smoking status, drinking status, BMI, physical activity, and family history of malignancy, and these differences were highly consistent with the known risk factors for esophageal cancer (8, 34).

Specifically, the EC patients had a higher proportion of male (59.0%), smoker (26.7%), drinker (32.4%), and individuals with a positive family history of malignancy (37.1%), along with the lowest BMI (22.21 ± 3.40) and the highest proportion of participants with education level of primary school and below (78.1%). The high-risk group had the highest proportion of Farmer (88.9%), the lowest proportion of married individuals (75.3%), and the highest level of sweating-type physical activity (representing moderate-to-high intensity activity, 63.0%). In contrast, the normal group had the highest BMI (26.26 ± 4.53) and the highest proportion of participants with education level of senior high school and above (18.5%).

These baseline differences suggest that the occurrence of EC is the result of the combined effects of genetic factors (family history of malignancy), behavioral factors (smoking status, drinking status), socioeconomic factors (education, occupation), and physiological status (BMI) (35, 36). This also provides a rational basis for adjusting these factors as covariates in the subsequent analysis of the association between immune indicators and EC grouping, ensuring the accuracy of the analysis of differences in immune indicators.

### 4.2 Immune indicator differences: biological mechanisms and association with EC progression

Through multi-dimensional statistical analysis, this study confirmed that EC grouping had a significant overall impact on 54 immune indicators (MANOVA, Pillai=0.585, *P* < 2.2×10^^−16^) and identified 12 key immune indicators that remained significantly different after FDR correction. These indicators included Tcm CD4^+^ T cells, Naive CD8^+^ T cells, Tem CD8^+^ T cells, VS-Td CD8^+^ T cells, FB CD8^+^ T cells, Th2 cells, Act NK cells, Plasma cells, Func mat Vδ1 cells, Im Reg B cells, Pre-naive cells, and Tc1 cells—covering cellular immunity, humoral immunity, and innate immunity. The differences in these indicators profoundly reflect the remodeling of immune function in the body at different risk stages of esophageal cancer, and their biological significance is closely related to the mechanism of EC development and progression (37, 38).

From the perspective of cellular immunity, the changes in T cell subsets were the most significant. As shown in **Table 1**, the levels of Th cells, Tem CD8^+^ T cells, and Th2 cells were the highest in EC patients; the high-risk group was dominated by Tc cells, Td CD8^+^ T cells, and Th1 cells; while the normal group showed an advantage in the level of Act NK cells. This difference suggests that during the occurrence of esophageal cancer, the body’s immune response undergoes a transformation from “defensive compensation” to “functional imbalance” (39): The high expression of Th1 cells and Tc cells in the high-risk group may represent a cellular immune defense initiated by the body against potential cancerous cells, attempting to enhance the tumor-clearing ability of Tc cells through Th1-type cytokines (such as IFN-γ) (40, 41). However, as the disease progresses to the EC stage, Th2 cells dominate over Th1 cells, leading to Th1/Th2 immune imbalance and inhibiting cellular immune function (9). At the same time, although the number of Tem CD8^+^ T cells increases, their function may be impaired by immunosuppressive factors (such as PD-L1) in the tumor microenvironment, forming “exhausted” T cells that cannot effectively clear tumor cells (42, 43). In addition, the level of Treg cells in EC patients was higher than that in the other two groups, which further aggravated the immunosuppressive state and created favorable conditions for the immune escape of tumor cells (44, 45).

In terms of innate immunity and humoral immunity, the normal group had the highest levels of Act NK cells and Vδ2 cells, while EC patients had significantly higher levels of Plasma cells and Func mat Vδ1 cells. As core effector cells of innate immunity, NK cells showed reduced activation level (reflected by Act NK cells) in high-risk group and EC patients, which indicates weakened innate immune killing ability and failure to promptly clear early abnormal cells. Vδ2 cells, as the main anti-tumor subset of γδT cells, their high expression in the normal group further confirms the defensive role of innate immunity against esophageal cancer, and the decrease in their levels may be an important marker of increased EC risk.

The high expression of Plasma cells in EC patients, although reflecting the activation of humoral immune response, may be due to the inability of the produced antibodies to specifically recognize tumor antigens or the formation of immune complexes that promote tumor progression. This suggests that humoral immunity may exhibit a “non-effective response” characteristic in esophageal cancer.

Notably, FB CD8^+^ T cells showed extremely strong robustness in stratified sensitivity analysis. Regardless of stratification by gender, age (<71 years, ≥71 years), education (primary school and below, junior high school, senior high school and above), or occupation (farmer, non-farmer), significant differences (*P* < 0.05) in this indicator among the three groups were maintained. As a functionally impaired T cell subset, changes in the level of FB CD8^+^ T cells may directly reflect the degree of decline in the body’s anti-tumor immune function, and it can be used as a potential “immune marker” for assessing EC risk, providing an important target for subsequent clinical transformation.

### 4.3 Predictive model efficacy and clinical application value

The neural network three-classification predictive model constructed based on the 12 key immune indicators mentioned above and 10 covariates (including gender, age, marital status, education, occupation, etc.) showed excellent discriminative efficacy in 10-fold cross-validation: the cross-validated overall accuracy was 81.2%, the Kappa coefficient was 0.709, and the AUC values for pairwise comparisons among the three populations all exceeded 0.9 (AUC = 0.927 for normal group vs. high-risk group, AUC = 0.914 for normal group vs. EC patients, and AUC = 0.905 for high-risk group vs. EC patients). This efficacy was significantly higher than that of traditional EC screening indicators (e.g., the sensitivity of esophageal mucosal biopsy is approximately 70%-80%).

From the perspective of clinical application, this model has three core values: First, it realizes “non-invasive risk stratification”. The detection of peripheral blood immune indicators relied on by the model only requires 5mL of fasting venous blood. Compared with the invasive operation of gastroscopy, it has higher patient acceptance and is more suitable for large-scale population screening for EC risk—especially for preliminary screening of high-risk group in high-incidence areas of esophageal cancer, which helps reduce the missed diagnosis rate. Second, it provides a “basis for dynamic monitoring”. The 12 key immune indicators (such as FB CD8^+^ T cells, Act NK cells, Th2 cells) can be used as dynamic monitoring indicators for EC risk progression. By regularly detecting changes in these indicators, the transformation trend of high-risk group to precancerous lesions or EC can be detected early, gaining time for interventional treatment. Third, it assists in “individualized treatment decision-making”. The combination of covariates (such as age, gender, smoking status) included in the model and immune indicators can help clinicians judge the immune function status of patients. For example, for patients with extremely low Act NK cells levels, combined treatment with NK cell activators can be considered to enhance the therapeutic effect, providing a reference for formulating individualized plans including immunotherapy for EC patients.

### 4.4 Study limitations and future directions

Although this study clarified the association between immune indicators and EC risk, it still has two limitations: First, the geographical concentration of sample sources. The study samples were mainly from high-incidence areas of EC such as Yanjin, Yuanyang, and Anyang Linzhou, as well as some community hospitals, which may lead to geographical selection bias. The applicability of the model in non-high-incidence areas needs further verification. Second, the cross-sectional nature of the study design. It is impossible to clarify the causal relationship between changes in immune indicators and the occurrence of esophageal cancer. For example, it cannot be determined whether the increase in Th2 cells leads to the occurrence of EC or the occurrence of EC induces an increase in Th2 cells.

Future studies can be carried out in two aspects: On the one hand, conduct multi-center, prospective cohort studies, expand the sample sources to non-high-incidence areas, dynamically track changes in key immune indicators (such as FB CD8^+^ T cells, Act NK cells) of high-risk group and the incidence outcomes of esophageal cancer, and verify the causal role of immune indicators. On the other hand, deepen mechanistic research. Focus on key indicators such as FB CD8^+^ T cells and Act NK cells to explore their regulatory pathways (e.g., the impact of the PD-1/PD-L1 signaling pathway on FB CD8^+^ T cells), provide experimental basis for the development of EC immunotherapy targets, and promote the transformation of research results from “risk assessment” to “therapeutic intervention”.

## 5 Conclusion

This study identified multiple immune characteristics with significant differences among populations at different EC risk levels using Analysis of Covariance (ANCOVA). Stratified sensitivity analysis confirmed the robustness of the key findings (e.g., functionally blocked CD8^+^ T cells, activated NK cells) across different demographic subgroups. Furthermore, the constructed machine learning model verified that combining these immune indicators with covariates can effectively distinguish between normal group, high-risk group, and EC patients, demonstrating high discriminative efficacy. This provides a potential basis for disease risk early warning and mechanism exploration.

## Data Availability

The original contributions presented in the study are included in the article/Supplementary Material. Further inquiries can be directed to the corresponding authors.
