# Supplementary material for: Differences in immune indicators among normal, high-risk, and esophageal cancer populations and development of a predictive model
Source: Front Immunol. 2026 Jan 30;17:1723700. doi: 10.3389/fimmu.2026.1723700 (PMC12901429; doi:10.3389/fimmu.2026.1723700)
Supplement: Supplementary file 1 [file Table1.docx]

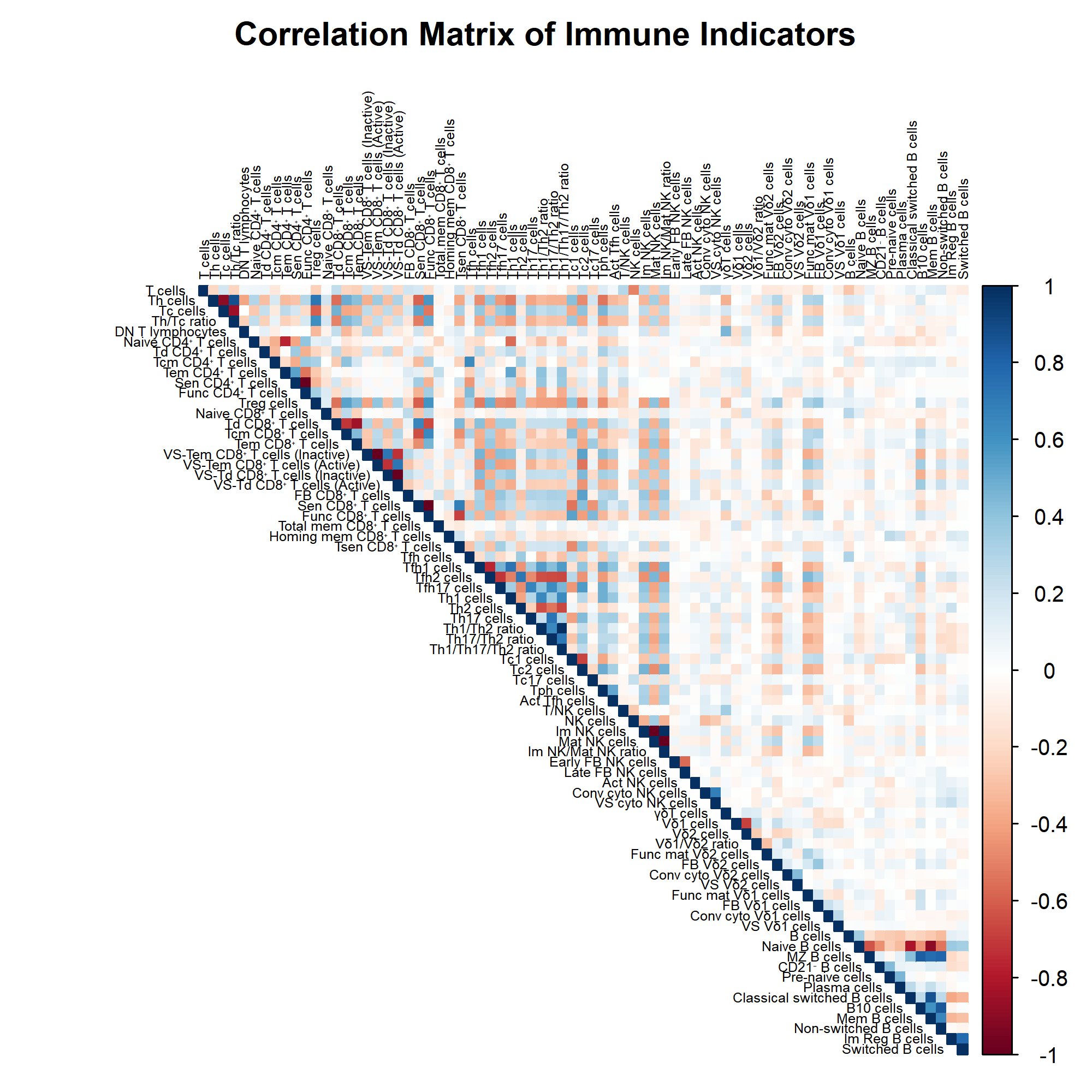


**Figure S1** Correlation Coefficient Heatmap of 75 Immune Indicators.

**Table S1** 75 Immune Indicators and Their Abbreviations.

| **Immune indicators** | **Abbreviations** |  | **Immune indicators** | **Abbreviations** |
| --- | --- | --- | --- | --- |
| T cells | T cells |  | Cytotoxic T cell 17 | Tc17 cells |
| Helper T cells | Th cells |  | Peripheral helper T cells | Tph cells |
| Cytotoxic T cells | Tc cells |  | Activated Tfh cells | Act Tfh cells |
| Th/Tc ratio | Th/Tc ratio |  | T/NK cells | T/NK cells |
| Double-negative T lymphocytes | DN T lymphocytes |  | Natural killer cells | NK cells |
| Naive CD4⁺ T cells | Naive CD4⁺ T cells |  | Immature NK cells | Im NK cells |
| Terminally differentiated CD4⁺ T cells | Td CD4⁺ T cells |  | Maturecon  NK cells | Mat NK cells |
| Central memory CD4⁺ T cells | Tcm CD4⁺ T cells |  | Immature NK cells/mature NK cells | Im NK/Mat NK ratio |
| Effector memory CD4⁺ T cells | Tem CD4⁺ T cells |  | Early functionally blocked NK cells | Early FB NK cells |
| Senescent CD4⁺ T cells | Sen CD4⁺ T cells |  | Late functionally blocked NK cells | Late FB NK cells |
| Functional CD4⁺ T cells | Func CD4⁺ T cells |  | Activated NK cells | Act NK cells |
| Regulatory T cells | Treg cells |  | Conventional cytotoxic NK cells | Conv cyto NK cells |
| Naive CD8⁺ T cells | Naive CD8⁺ T cells |  | Virus-specific cytotoxic NK cells | VS cyto NK cells |
| Terminally differentiated CD8⁺ T cells | Td CD8⁺ T cells |  | γδ T cells | γδT cells |
| Central memory CD8⁺ T cells | Tcm CD8⁺ T cells |  | Vδ1 cells | Vδ1 cells |
| Effector memory CD8⁺ T cells | Tem CD8⁺ T cells |  | Vδ2 cells | Vδ2 cells |
| Virus-specific effector memory CD8⁺ T cells with inactive expression | VS-Tem CD8⁺ T cells (Inactive) |  | Vδ1/Vδ2 ratio | Vδ1/Vδ2 ratio |
| Virus-specific effector memory CD8⁺ T cells with active expression | VS-Tem CD8⁺ T cells (Active) |  | Functionally mature Vδ2 cells | Func mat Vδ2 cells |
| Virus-specific terminally differentiated CD8⁺ T cells with inactive expression | VS-Td CD8⁺ T cells (Inactive) |  | Functionally blocked Vδ2 cells | FB Vδ2 cells |
| Virus-specific terminally differentiated CD8⁺ T cells with active expression | VS-Td CD8⁺ T cells (Active) |  | Conventional cytotoxic Vδ2 cells | Conv cyto Vδ2 cells |
| Functionally blocked CD8⁺ T cells | FB CD8⁺ T cells |  | Virus-specific Vδ2 cells | VS Vδ2 cells |
| Senescent CD8⁺ T cells | Sen CD8⁺ T cells |  | Functionally mature Vδ1 cells | Func mat Vδ1 cells |
| Functional CD8⁺ T cells | Func CD8⁺ T cells |  | Functionally blocked Vδ1 cells | FB Vδ1 cells |
| Total memory CD8⁺ T cells | Total mem CD8⁺ T cells |  | Conventional cytotoxic Vδ1 cells | Conv cyto Vδ1 cells |
| Homing memory CD8⁺ T cells | Homing mem CD8⁺ T cells |  | Virus-specific Vδ1 cells | VS Vδ1 cells |
| Terminally senescent CD8⁺ T cells | Tsen CD8⁺ T cells |  | B cells | B cells |
| Follicular helper T cells | Tfh cells |  | Naive B cells | Naive B cells |
| Follicular helper T cells 1 | Tfh1 cells |  | Marginal zone B cells | MZ B cells |
| Follicular helper T cells 2 | Tfh2 cells |  | CD21⁻ B cells | CD21⁻ B cells |
| Follicular helper T cells 17 | Tfh17 cells |  | Pre-naive cells | Pre-naive cells |
| T helper 1 cells | Th1 cells |  | Plasma cells | Plasma cells |
| T helper 2 cells | Th2 cells |  | Classical switched B cells | Classical switched B cells |
| T helper 17 cells | Th17 cells |  | B10 cells | B10 cells |
| Th1/Th2 ratio | Th1/Th2 ratio |  | Memory B cells | Mem B cells |
| Th17/Th2 ratio | Th17/Th2 ratio |  | Non-switched B cells | Non-switched B cells |
| Th1/Th17/Th2 ratio | Th1/Th17/Th2 ratio |  | Immature regulatory B cells | Im Reg B cells |
| Cytotoxic T cell 1 | Tc1 cells |  | Switched B cells | Switched B cells |
| Cytotoxic T cell 2 | Tc2 cells |  |  |  |

**Table S2** Stratified Sensitivity Analysis (Stratified by Gender).

| **ImmuneMetrics** |  | **Gender** | | | | | | |
| --- | --- | --- | --- | --- | --- | --- | --- | --- |
|  | **Man** | | |  | | **Woman** | | |
|  | **F** | ***P*** | ***P'*** |  | **F** | | ***P*** | ***P'*** |
| Tcells | 2.271 | 0.133 | 0.313 |  | 3.370 | | 0.068 | 0.152 |
| Cytotoxic Tcells | 2.713 | 0.101 | 0.260 |  | 5.307 | | 0.022 | 0.085 |
| DNTlymphocytes | 0.082 | 0.775 | 0.932 |  | 0.429 | | 0.513 | 0.771 |
| Naïve CD4⁺T cells | 4.280 | 0.040 | 0.143 |  | 0.591 | | 0.443 | 0.703 |
| Td CD4⁺ T cells | 0.873 | 0.351 | 0.575 |  | 0.015 | | 0.902 | 0.919 |
| Tcm CD4⁺ T cells | 22.149 | 0.000 | 0.000 |  | 3.785 | | 0.053 | 0.143 |
| Sen CD4⁺ T cells | 1.144 | 0.286 | 0.483 |  | 0.150 | | 0.699 | 0.793 |
| Naïve CD8⁺ T cells | 4.179 | 0.042 | 0.143 |  | 13.479 | | 0.000 | 0.003 |
| Tcm CD8⁺ T cells | 6.519 | 0.011 | 0.056 |  | 3.007 | | 0.084 | 0.181 |
| Tem CD8⁺ T cells | 20.930 | 0.000 | 0.000 |  | 22.870 | | 0.000 | 0.000 |
| VS-Td CD8⁺ T cells | 1.398 | 0.238 | 0.429 |  | 16.460 | | 0.000 | 0.001 |
| FB CD8⁺ T cells | 29.157 | 0.000 | 0.000 |  | 29.826 | | 0.000 | 0.000 |
| Sen CD8⁺ T cells | 4.727 | 0.031 | 0.128 |  | 0.093 | | 0.760 | 0.821 |
| Total mem CD8⁺ T cells | 1.672 | 0.197 | 0.386 |  | 0.356 | | 0.551 | 0.783 |
| Homing mem CD8⁺ T cells | 2.943 | 0.088 | 0.250 |  | 4.450 | | 0.036 | 0.108 |
| Tsen CD8⁺ T cells | 8.715 | 0.004 | 0.024 |  | 3.498 | | 0.063 | 0.152 |
| Tfh cells | 2.423 | 0.121 | 0.297 |  | 0.109 | | 0.741 | 0.817 |
| Tfh17 cells | 2.713 | 0.101 | 0.260 |  | 5.752 | | 0.017 | 0.072 |
| Th1 cells | 1.144 | 0.286 | 0.483 |  | 6.292 | | 0.013 | 0.058 |
| Th2 cells | 9.705 | 0.002 | 0.019 |  | 28.285 | | 0.000 | 0.000 |
| Th17 cells | 0.338 | 0.562 | 0.867 |  | 1.433 | | 0.232 | 0.433 |
| Th1/Th2 ratio | 1.869 | 0.173 | 0.360 |  | 8.829 | | 0.003 | 0.018 |
| Tc1 cells | 0.000 | 0.988 | 0.988 |  | 1.821 | | 0.179 | 0.344 |
| Tc2 cells | 6.643 | 0.011 | 0.056 |  | 2.950 | | 0.087 | 0.181 |
| Tc17 cells | 3.837 | 0.052 | 0.155 |  | 0.278 | | 0.598 | 0.793 |
| Tph cells | 4.319 | 0.039 | 0.143 |  | 0.229 | | 0.633 | 0.793 |
| Act Tfh cells | 8.740 | 0.003 | 0.024 |  | 0.214 | | 0.644 | 0.793 |
| T/NK cells | 1.903 | 0.169 | 0.360 |  | 0.038 | | 0.846 | 0.878 |
| NK cells | 0.122 | 0.727 | 0.932 |  | 5.090 | | 0.025 | 0.090 |
| Im NK/Mat NK ratio | 7.597 | 0.006 | 0.038 |  | 4.569 | | 0.034 | 0.108 |
| Early FB NK cells | 0.146 | 0.703 | 0.932 |  | 1.299 | | 0.256 | 0.460 |
| Late FB NK cells | 0.106 | 0.745 | 0.932 |  | 0.295 | | 0.588 | 0.793 |
| Act NK cells | 13.015 | 0.000 | 0.005 |  | 28.037 | | 0.000 | 0.000 |
| Conv cyto NK cells | 1.646 | 0.201 | 0.386 |  | 3.893 | | 0.050 | 0.141 |
| VS cyto NK cells | 0.046 | 0.831 | 0.955 |  | 0.213 | | 0.645 | 0.793 |
| γδT cells | 0.003 | 0.958 | 0.981 |  | 0.427 | | 0.514 | 0.771 |
| Vδ1 cells | 0.015 | 0.902 | 0.975 |  | 0.640 | | 0.424 | 0.694 |
| Vδ2 cells | 0.183 | 0.669 | 0.932 |  | 4.450 | | 0.036 | 0.108 |
| Vδ1/Vδ2ratio | 1.599 | 0.208 | 0.386 |  | 0.054 | | 0.817 | 0.865 |
| Func mat Vδ2 cells | 0.492 | 0.484 | 0.769 |  | 7.794 | | 0.006 | 0.028 |
| FB Vδ2 cells | 0.012 | 0.912 | 0.975 |  | 0.862 | | 0.354 | 0.613 |
| Conv cyto Vδ2 cells | 0.002 | 0.963 | 0.981 |  | 0.202 | | 0.654 | 0.793 |
| VS Vδ2 cells | 0.028 | 0.868 | 0.975 |  | 0.167 | | 0.683 | 0.793 |
| Func mat Vδ1 cells | 4.821 | 0.029 | 0.128 |  | 11.075 | | 0.001 | 0.008 |
| FB Vδ1 cells | 3.956 | 0.048 | 0.153 |  | 0.830 | | 0.363 | 0.613 |
| Conv cyto Vδ1 cells | 0.058 | 0.810 | 0.951 |  | 0.000 | | 0.996 | 0.996 |
| VS Vδ1 cells | 0.092 | 0.762 | 0.932 |  | 0.356 | | 0.551 | 0.783 |
| B cells | 0.010 | 0.921 | 0.975 |  | 0.170 | | 0.680 | 0.793 |
| MZ Bcells | 0.081 | 0.776 | 0.932 |  | 3.635 | | 0.058 | 0.149 |
| CD21-Bcells | 0.185 | 0.668 | 0.932 |  | 3.412 | | 0.066 | 0.152 |
| Pre-naïve cells | 1.940 | 0.165 | 0.360 |  | 8.903 | | 0.003 | 0.018 |
| Plasma cells | 10.130 | 0.002 | 0.018 |  | 2.725 | | 0.100 | 0.200 |
| Class-switched Bcells | 0.283 | 0.595 | 0.893 |  | 0.144 | | 0.705 | 0.793 |
| Im Reg Bcells | 0.083 | 0.773 | 0.932 |  | 10.244 | | 0.002 | 0.011 |

**Table S3** Stratified Sensitivity Analysis (Stratified by Education).

| **ImmuneMetrics** | **Education** | | | | | | | | | | | |  |
| --- | --- | --- | --- | --- | --- | --- | --- | --- | --- | --- | --- | --- | --- |
|  | **Primary school and below** | | | |  | **Junior high school** | | |  | **Senior high school and above** | | | |
|  | **F** | | ***P*** | ***P'*** |  | **F** | ***P*** | ***P'*** |  | **F** | ***P*** | ***P'*** |  |
| T cells | 3.556 | | 0.061 | 0.131 |  | 0.213 | 0.645 | 0.774 |  | 1.610 | 0.210 | 0.843 |  |
| CytotoxicTcells | 10.254 | | 0.002 | 0.014 |  | 0.249 | 0.619 | 0.774 |  | 0.838 | 0.364 | 0.854 |  |
| DNT lymphocytes | | 0.073 | 0.788 | 0.858 |  | 0.706 | 0.403 | 0.767 |  | 1.685 | 0.200 | 0.843 |  |
| Naïve CD4⁺ T cells | 1.651 | | 0.200 | 0.386 |  | 1.508 | 0.222 | 0.545 |  | 0.040 | 0.842 | 0.939 |  |
| Td CD4⁺ T cells | 0.195 | | 0.659 | 0.792 |  | 0.331 | 0.566 | 0.767 |  | 0.822 | 0.369 | 0.854 |  |
| Tcm CD4⁺ T cells | 4.331 | | 0.038 | 0.090 |  | 10.753 | 0.001 | 0.018 |  | 0.765 | 0.386 | 0.854 |  |
| Sen CD4⁺ T cells | 0.194 | | 0.660 | 0.792 |  | 0.221 | 0.639 | 0.774 |  | 0.059 | 0.810 | 0.939 |  |
| Naïve CD8⁺ T cells | 8.062 | | 0.005 | 0.033 |  | 9.412 | 0.003 | 0.029 |  | 1.313 | 0.257 | 0.854 |  |
| Tcm CD8⁺ T cells | 0.049 | | 0.824 | 0.873 |  | 1.262 | 0.263 | 0.609 |  | 0.794 | 0.377 | 0.854 |  |
| Tem CD8⁺ T cells | 25.405 | | 0.000 | 0.000 |  | 4.262 | 0.041 | 0.244 |  | 2.092 | 0.154 | 0.843 |  |
| VS-Td CD8⁺ T cells | 8.027 | | 0.005 | 0.033 |  | 4.472 | 0.037 | 0.244 |  | 2.795 | 0.101 | 0.843 |  |
| FB CD8⁺ T cells | 18.097 | | 0.000 | 0.001 |  | 29.955 | 0.000 | 0.000 |  | 13.542 | 0.001 | 0.031 |  |
| Sen CD8⁺ T cells | 0.784 | | 0.377 | 0.616 |  | 0.366 | 0.546 | 0.767 |  | 0.032 | 0.859 | 0.939 |  |
| Total mem CD8⁺ T cells | 1.258 | | 0.263 | 0.458 |  | 0.059 | 0.808 | 0.873 |  | 0.020 | 0.887 | 0.939 |  |
| Homing mem CD8⁺ T cells | 4.668 | | 0.032 | 0.090 |  | 2.315 | 0.131 | 0.442 |  | 0.004 | 0.952 | 0.970 |  |
| Tsen CD8⁺ T cells | 4.353 | | 0.038 | 0.090 |  | 0.075 | 0.784 | 0.873 |  | 1.961 | 0.168 | 0.843 |  |
| Tfh cells | 0.679 | | 0.411 | 0.628 |  | 3.929 | 0.050 | 0.244 |  | 0.293 | 0.591 | 0.939 |  |
| Tfh17 cells | 5.715 | | 0.018 | 0.079 |  | 2.488 | 0.117 | 0.428 |  | 3.751 | 0.058 | 0.789 |  |
| Th1 cells | 5.361 | | 0.021 | 0.089 |  | 0.237 | 0.627 | 0.774 |  | 0.080 | 0.779 | 0.939 |  |
| Th2 cells | 13.003 | | 0.000 | 0.004 |  | 14.792 | 0.000 | 0.004 |  | 0.757 | 0.389 | 0.854 |  |
| Th17 cells | 0.142 | | 0.707 | 0.795 |  | 1.146 | 0.287 | 0.609 |  | 0.950 | 0.335 | 0.854 |  |
| Th1/Th2 ratio | 4.521 | | 0.034 | 0.090 |  | 4.026 | 0.047 | 0.244 |  | 0.109 | 0.743 | 0.939 |  |
| Tc1 cells | 4.648 | | 0.032 | 0.090 |  | 0.022 | 0.883 | 0.935 |  | 0.401 | 0.530 | 0.939 |  |
| Tc2 cells | 1.445 | | 0.230 | 0.415 |  | 2.467 | 0.119 | 0.428 |  | 1.553 | 0.219 | 0.843 |  |
| Tc17 cells | 3.622 | | 0.058 | 0.131 |  | 0.000 | 0.991 | 0.992 |  | 1.237 | 0.271 | 0.854 |  |
| Tph cells | 1.126 | | 0.290 | 0.489 |  | 0.352 | 0.554 | 0.767 |  | 0.087 | 0.770 | 0.939 |  |
| Act Tfh cells | 6.966 | | 0.009 | 0.048 |  | 0.003 | 0.956 | 0.992 |  | 2.065 | 0.157 | 0.843 |  |
| T/NK cells | 0.624 | | 0.430 | 0.628 |  | 1.724 | 0.192 | 0.518 |  | 0.260 | 0.613 | 0.939 |  |
| NK cells | 0.634 | | 0.427 | 0.628 |  | 5.351 | 0.022 | 0.173 |  | 0.161 | 0.690 | 0.939 |  |
| Im NK/Mat NK ratio | 5.069 | | 0.025 | 0.090 |  | 2.141 | 0.146 | 0.464 |  | 0.708 | 0.404 | 0.854 |  |
| Early FB NK cells | 0.503 | | 0.479 | 0.663 |  | 0.442 | 0.507 | 0.767 |  | 0.372 | 0.545 | 0.939 |  |
| Late FB NK cells | 0.509 | | 0.476 | 0.663 |  | 0.355 | 0.553 | 0.767 |  | 0.689 | 0.411 | 0.854 |  |
| Act NK cells | 13.021 | | 0.000 | 0.004 |  | 37.590 | 0.000 | 0.000 |  | 5.408 | 0.024 | 0.435 |  |
| Conv cyto NK cells | 4.885 | | 0.028 | 0.090 |  | 0.397 | 0.530 | 0.767 |  | 0.541 | 0.465 | 0.906 |  |
| VS cyto NK cells | 2.680 | | 0.103 | 0.214 |  | 7.474 | 0.007 | 0.065 |  | 2.638 | 0.111 | 0.843 |  |
| Γδ T cells | 0.024 | | 0.877 | 0.911 |  | 0.341 | 0.560 | 0.767 |  | 0.048 | 0.827 | 0.939 |  |
| Vδ1 cells | 0.068 | | 0.795 | 0.858 |  | 0.328 | 0.568 | 0.767 |  | 0.014 | 0.906 | 0.941 |  |
| Vδ2 cells | 0.475 | | 0.491 | 0.663 |  | 1.209 | 0.274 | 0.609 |  | 0.530 | 0.470 | 0.906 |  |
| Vδ1/Vδ2 ratio | 0.171 | | 0.679 | 0.795 |  | 1.114 | 0.293 | 0.609 |  | 0.099 | 0.755 | 0.939 |  |
| Func mat Vδ2 cells | 5.965 | | 0.015 | 0.075 |  | 0.337 | 0.563 | 0.767 |  | 0.687 | 0.411 | 0.854 |  |
| FB Vδ2 cells | 0.271 | | 0.603 | 0.776 |  | 0.140 | 0.709 | 0.815 |  | 0.000 | 0.987 | 0.987 |  |
| Conv cyto Vδ2 cells | 0.623 | | 0.431 | 0.628 |  | 0.164 | 0.686 | 0.806 |  | 0.052 | 0.820 | 0.939 |  |
| VS Vδ2 cells | 0.445 | | 0.505 | 0.665 |  | 0.266 | 0.607 | 0.774 |  | 0.240 | 0.626 | 0.939 |  |
| Func mat Vδ1 cells | 19.508 | | 0.000 | 0.000 |  | 1.998 | 0.160 | 0.468 |  | 0.034 | 0.854 | 0.939 |  |
| FB Vδ1cells | 0.007 | | 0.931 | 0.949 |  | 0.572 | 0.451 | 0.767 |  | 0.312 | 0.579 | 0.939 |  |
| Conv cyto Vδ1 cells | 0.142 | | 0.706 | 0.795 |  | 0.060 | 0.808 | 0.873 |  | 1.729 | 0.195 | 0.843 |  |
| VS Vδ1 cells | 0.001 | | 0.979 | 0.979 |  | 0.601 | 0.440 | 0.767 |  | 1.312 | 0.257 | 0.854 |  |
| B cells | 0.227 | | 0.634 | 0.792 |  | 0.000 | 0.992 | 0.992 |  | 0.060 | 0.807 | 0.939 |  |
| MZ B cells | 4.367 | | 0.038 | 0.090 |  | 1.559 | 0.214 | 0.545 |  | 0.100 | 0.753 | 0.939 |  |
| CD21-B cells | 1.493 | | 0.223 | 0.415 |  | 0.803 | 0.372 | 0.744 |  | 0.115 | 0.736 | 0.939 |  |
| Pre-naïve cells | 4.456 | | 0.036 | 0.090 |  | 1.956 | 0.165 | 0.468 |  | 6.834 | 0.012 | 0.318 |  |
| Plasma cells | 7.820 | | 0.006 | 0.033 |  | 3.194 | 0.076 | 0.318 |  | 3.261 | 0.077 | 0.831 |  |
| Class-switched B cells | 1.694 | | 0.194 | 0.386 |  | 0.555 | 0.458 | 0.767 |  | 0.056 | 0.813 | 0.939 |  |
| Im Reg Bcells | 4.525 | | 0.034 | 0.090 |  | 3.592 | 0.061 | 0.272 |  | 0.020 | 0.887 | 0.939 |  |

**Table S4** Stratified Sensitivity Analysis (Stratified by Occupation).

| **ImmuneMetrics** | **Occupation** | | | | | | | |
| --- | --- | --- | --- | --- | --- | --- | --- | --- |
|  | **Farmer** | | |  | | **Non-farmer** | | |
|  | **F** | ***P*** | ***P'*** |  | **F** | | ***P*** | ***P'*** |
| T cells | 0.664 | 0.416 | 0.647 |  | 3.840 | | 0.053 | 0.151 |
| Cytotoxic T cells | 5.523 | 0.019 | 0.080 |  | 0.501 | | 0.481 | 0.666 |
| DNT lymphocytes | 1.437 | 0.231 | 0.446 |  | 0.820 | | 0.367 | 0.551 |
| Naïve CD4⁺ T cells | 1.845 | 0.175 | 0.397 |  | 1.120 | | 0.293 | 0.465 |
| Td CD4⁺ T cells | 0.076 | 0.783 | 0.900 |  | 0.468 | | 0.496 | 0.669 |
| Tcm CD4⁺ T cells | 7.227 | 0.008 | 0.037 |  | 10.478 | | 0.002 | 0.014 |
| Sen CD4⁺ T cells | 0.137 | 0.711 | 0.892 |  | 1.338 | | 0.250 | 0.436 |
| Naïve CD8⁺ T cells | 5.582 | 0.019 | 0.080 |  | 8.033 | | 0.006 | 0.033 |
| Tcm CD8⁺ T cells | 1.136 | 0.287 | 0.500 |  | 5.705 | | 0.019 | 0.067 |
| Tem CD8⁺ T cells | 12.410 | 0.000 | 0.005 |  | 33.508 | | 0.000 | 0.000 |
| VS-Td CD8⁺ T cells | 18.053 | 0.000 | 0.001 |  | 0.212 | | 0.647 | 0.759 |
| FB CD8⁺ T cells | 18.342 | 0.000 | 0.001 |  | 27.216 | | 0.000 | 0.000 |
| Sen CD8⁺ T cells | 0.015 | 0.902 | 0.937 |  | 3.065 | | 0.083 | 0.189 |
| Total mem CD8⁺ T cells | 4.323 | 0.038 | 0.138 |  | 0.410 | | 0.524 | 0.676 |
| Homing mem CD8⁺ T cells | 7.349 | 0.007 | 0.037 |  | 0.266 | | 0.607 | 0.729 |
| Tsen CD8⁺ T cells | 1.724 | 0.190 | 0.397 |  | 10.239 | | 0.002 | 0.014 |
| Tfh cells | 0.284 | 0.594 | 0.783 |  | 3.850 | | 0.053 | 0.151 |
| Tfh17 cells | 0.653 | 0.419 | 0.647 |  | 5.615 | | 0.020 | 0.067 |
| Th1 cells | 2.603 | 0.108 | 0.303 |  | 2.674 | | 0.105 | 0.228 |
| Th2 cells | 11.912 | 0.001 | 0.006 |  | 27.930 | | 0.000 | 0.000 |
| Th17 cells | 2.534 | 0.112 | 0.303 |  | 6.709 | | 0.011 | 0.043 |
| Th1/Th2 ratio | 2.396 | 0.123 | 0.315 |  | 3.052 | | 0.084 | 0.189 |
| Tc1 cells | 9.870 | 0.002 | 0.012 |  | 1.597 | | 0.209 | 0.390 |
| Tc2 cells | 0.036 | 0.849 | 0.928 |  | 8.592 | | 0.004 | 0.029 |
| Tc17 cells | 1.180 | 0.278 | 0.500 |  | 0.050 | | 0.824 | 0.872 |
| Tph cells | 0.522 | 0.470 | 0.687 |  | 3.292 | | 0.073 | 0.187 |
| Act Tfh cells | 2.195 | 0.139 | 0.342 |  | 6.999 | | 0.010 | 0.040 |
| T/NK cells | 1.050 | 0.306 | 0.517 |  | 0.028 | | 0.867 | 0.884 |
| NK cells | 1.360 | 0.244 | 0.455 |  | 1.196 | | 0.277 | 0.465 |
| Im NK/Mat NK ratio | 1.785 | 0.182 | 0.397 |  | 17.217 | | 0.000 | 0.001 |
| Early FB NK cells | 0.855 | 0.356 | 0.582 |  | 0.157 | | 0.693 | 0.796 |
| Late FB NK cells | 0.015 | 0.901 | 0.937 |  | 0.634 | | 0.428 | 0.625 |
| Act NK cells | 15.476 | 0.000 | 0.002 |  | 26.494 | | 0.000 | 0.000 |
| Conv cyto NK cells | 5.345 | 0.021 | 0.083 |  | 1.382 | | 0.243 | 0.436 |
| VS cyto NK cells | 1.700 | 0.193 | 0.397 |  | 1.812 | | 0.182 | 0.360 |
| γδT cells | 0.076 | 0.783 | 0.900 |  | 1.848 | | 0.177 | 0.360 |
| Vδ1 cells | 0.574 | 0.449 | 0.674 |  | 1.768 | | 0.187 | 0.360 |
| Vδ2 cells | 0.032 | 0.859 | 0.928 |  | 0.406 | | 0.525 | 0.676 |
| Vδ1/Vδ2 ratio | 1.659 | 0.199 | 0.397 |  | 0.600 | | 0.440 | 0.626 |
| Func mat Vδ2 cells | 0.464 | 0.496 | 0.691 |  | 7.698 | | 0.007 | 0.033 |
| FB Vδ2 cells | 0.177 | 0.674 | 0.867 |  | 0.268 | | 0.606 | 0.729 |
| Conv cyto Vδ2 cells | 0.009 | 0.924 | 0.942 |  | 7.079 | | 0.009 | 0.040 |
| VS Vδ2 cells | 0.120 | 0.729 | 0.892 |  | 0.034 | | 0.855 | 0.884 |
| Func mat Vδ1 cells | 3.380 | 0.067 | 0.212 |  | 7.847 | | 0.006 | 0.033 |
| FB Vδ1 cells | 0.107 | 0.744 | 0.892 |  | 3.144 | | 0.079 | 0.189 |
| Conv cyto Vδ1 cells | 0.000 | 0.998 | 0.998 |  | 0.280 | | 0.598 | 0.729 |
| VS Vδ1 cells | 0.432 | 0.511 | 0.691 |  | 5.049 | | 0.027 | 0.086 |
| B cells | 0.431 | 0.512 | 0.691 |  | 0.134 | | 0.715 | 0.805 |
| MZ B cells | 4.179 | 0.042 | 0.141 |  | 0.057 | | 0.812 | 0.872 |
| CD21-Bcells | 2.955 | 0.087 | 0.260 |  | 0.085 | | 0.772 | 0.851 |
| Pre-naïve cells | 11.238 | 0.001 | 0.007 |  | 0.939 | | 0.335 | 0.517 |
| Plasma cells | 12.542 | 0.000 | 0.005 |  | 3.605 | | 0.061 | 0.164 |
| Class-switched B cells | 0.042 | 0.837 | 0.928 |  | 1.133 | | 0.290 | 0.465 |
| Im Reg Bcells | 8.275 | 0.004 | 0.026 |  | 0.007 | | 0.935 | 0.935 |

**Table S5** Stratified Sensitivity Analysis (Stratified by Age).

| **ImmuneMetrics** | **Age** | | | | | | | |
| --- | --- | --- | --- | --- | --- | --- | --- | --- |
|  | **＜71 years** | | |  | | **≥71** **years** | | |
|  | **F** | ***P*** | ***P'*** |  | **F** | | ***P*** | ***P'*** |
| T cells | 2.165 | 0.143 | 0.308 |  | 3.738 | | 0.055 | 0.198 |
| Cytotoxic T cells | 1.150 | 0.285 | 0.513 |  | 5.678 | | 0.018 | 0.097 |
| DNT lymphocytes | 0.601 | 0.439 | 0.628 |  | 3.722 | | 0.055 | 0.198 |
| Naïve CD4⁺ T cells | 6.480 | 0.012 | 0.055 |  | 0.457 | | 0.500 | 0.642 |
| Td CD4⁺ T cells | 0.004 | 0.948 | 0.948 |  | 0.037 | | 0.848 | 0.897 |
| Tcm CD4⁺ T cells | 9.098 | 0.003 | 0.017 |  | 9.188 | | 0.003 | 0.016 |
| Sen CD4⁺ T cells | 0.835 | 0.362 | 0.592 |  | 0.859 | | 0.355 | 0.504 |
| Naïve CD8⁺ T cells | 6.240 | 0.013 | 0.055 |  | 17.528 | | 0.000 | 0.001 |
| Tcm CD8⁺ T cells | 0.958 | 0.329 | 0.558 |  | 0.000 | | 0.998 | 0.998 |
| Tem CD8⁺ T cells | 11.277 | 0.001 | 0.006 |  | 32.373 | | 0.000 | 0.000 |
| VS-Td CD8⁺ T cells | 4.861 | 0.029 | 0.096 |  | 9.718 | | 0.002 | 0.016 |
| FB CD8⁺ T cells | 30.078 | 0.000 | 0.000 |  | 28.410 | | 0.000 | 0.000 |
| Sen CD8⁺ T cells | 2.026 | 0.156 | 0.312 |  | 0.081 | | 0.776 | 0.861 |
| Total mem CD8⁺ T cells | 0.032 | 0.858 | 0.945 |  | 2.677 | | 0.103 | 0.276 |
| Homing mem CD8⁺ T cells | 3.111 | 0.079 | 0.214 |  | 3.839 | | 0.051 | 0.198 |
| Tsen CD8⁺ T cells | 11.820 | 0.001 | 0.006 |  | 0.952 | | 0.330 | 0.504 |
| Tfh cells | 0.100 | 0.752 | 0.902 |  | 1.315 | | 0.253 | 0.455 |
| Tfh17 cells | 1.960 | 0.163 | 0.314 |  | 5.403 | | 0.021 | 0.103 |
| Th1 cells | 2.088 | 0.150 | 0.312 |  | 3.327 | | 0.070 | 0.221 |
| Th2 cells | 14.175 | 0.000 | 0.002 |  | 19.038 | | 0.000 | 0.000 |
| Th17 cells | 0.248 | 0.619 | 0.760 |  | 1.851 | | 0.175 | 0.385 |
| Th1/Th2 ratio | 2.170 | 0.142 | 0.308 |  | 9.267 | | 0.003 | 0.016 |
| Tc1 cells | 0.796 | 0.373 | 0.593 |  | 0.917 | | 0.339 | 0.504 |
| Tc2 cells | 6.289 | 0.013 | 0.055 |  | 2.357 | | 0.126 | 0.310 |
| Tc17 cells | 3.112 | 0.079 | 0.214 |  | 0.077 | | 0.781 | 0.861 |
| Tph cells | 2.602 | 0.108 | 0.278 |  | 0.441 | | 0.507 | 0.642 |
| Act Tfh cells | 8.799 | 0.003 | 0.018 |  | 0.107 | | 0.744 | 0.855 |
| T/NK cells | 0.035 | 0.851 | 0.945 |  | 0.869 | | 0.352 | 0.504 |
| NK cells | 0.005 | 0.944 | 0.948 |  | 2.616 | | 0.107 | 0.276 |
| Im NK/Mat NK ratio | 11.503 | 0.001 | 0.006 |  | 3.126 | | 0.078 | 0.223 |
| Early FB NK cells | 0.063 | 0.802 | 0.942 |  | 0.231 | | 0.632 | 0.758 |
| Late FB NK cells | 0.623 | 0.431 | 0.628 |  | 2.235 | | 0.136 | 0.320 |
| Act NK cells | 22.285 | 0.000 | 0.000 |  | 16.606 | | 0.000 | 0.001 |
| Conv cyto NK cells | 2.483 | 0.117 | 0.286 |  | 3.461 | | 0.064 | 0.217 |
| VS cyto NK cells | 0.564 | 0.454 | 0.628 |  | 1.447 | | 0.230 | 0.432 |
| γδT cells | 2.186 | 0.141 | 0.308 |  | 0.925 | | 0.337 | 0.504 |
| Vδ1 cells | 0.040 | 0.841 | 0.945 |  | 0.375 | | 0.541 | 0.664 |
| Vδ2 cells | 0.349 | 0.555 | 0.715 |  | 1.705 | | 0.193 | 0.386 |
| Vδ1/Vδ2 ratio | 0.611 | 0.435 | 0.628 |  | 1.766 | | 0.185 | 0.385 |
| Func mat Vδ2 cells | 18.006 | 0.000 | 0.001 |  | 0.433 | | 0.511 | 0.642 |
| FB Vδ2 cells | 0.011 | 0.917 | 0.948 |  | 0.447 | | 0.505 | 0.642 |
| Conv cyto Vδ2 cells | 0.011 | 0.915 | 0.948 |  | 0.003 | | 0.958 | 0.976 |
| VS Vδ2 cells | 0.586 | 0.445 | 0.628 |  | 0.473 | | 0.492 | 0.642 |
| Func mat Vδ1 cells | 4.431 | 0.037 | 0.115 |  | 15.185 | | 0.000 | 0.001 |
| FB Vδ1 cells | 4.968 | 0.027 | 0.096 |  | 0.914 | | 0.340 | 0.504 |
| Conv cyto Vδ1 cells | 0.347 | 0.556 | 0.715 |  | 0.055 | | 0.815 | 0.880 |
| VS Vδ1 cells | 0.952 | 0.330 | 0.558 |  | 0.007 | | 0.935 | 0.971 |
| B cells | 1.186 | 0.277 | 0.513 |  | 0.131 | | 0.718 | 0.842 |
| MZ B cells | 0.255 | 0.614 | 0.760 |  | 3.227 | | 0.074 | 0.221 |
| CD21-B cells | 0.368 | 0.545 | 0.715 |  | 1.438 | | 0.232 | 0.432 |
| Pre-naïve cells | 4.882 | 0.028 | 0.096 |  | 4.318 | | 0.039 | 0.175 |
| Plasma cells | 15.414 | 0.000 | 0.002 |  | 0.953 | | 0.330 | 0.504 |
| Class-switched B cells | 0.014 | 0.906 | 0.948 |  | 0.879 | | 0.350 | 0.504 |
| Im Reg B cells | 4.341 | 0.038 | 0.115 |  | 1.798 | | 0.181 | 0.385 |
